# Parents’ experiences of complementary feeding among a United Kingdom culturally diverse and deprived community

**DOI:** 10.1111/mcn.13108

**Published:** 2020-11-09

**Authors:** Erica Jane Cook, Faye Caroline Powell, Nasreen Ali, Catrin Penn‐Jones, Bertha Ochieng, Gurch Randhawa

**Affiliations:** ^1^ School of Psychology University of Bedfordshire Luton UK; ^2^ Institute for Health Research University of Bedfordshire Luton UK; ^3^ Faculty of Health and Life Sciences De Montfort University Leicester UK

**Keywords:** complementary feeding, cultural factors, deprivation, ethnicity, fathers, feeding practices, weaning

## Abstract

Complementary feeding practices and adherence to health recommendations are influenced by a range of different and often interrelating factors such as socio‐economic and cultural factors. However, the factors underlying these associations are often complex with less awareness of how complementary feeding approaches vary across the UK’s diverse population. This paper describes a qualitative investigation undertaken in a deprived and culturally diverse community in the UK which aimed to explore parents’ knowledge, beliefs and practices of complementary feeding. One hundred and ten mothers and fathers, self‐identified as being White British, Pakistani, Bangladeshi, Black African/Caribbean or Polish took part in twenty‐four focus group discussions, organised by age group, sex and ethnicity.

The findings revealed that most parents initiated complementary feeding before the World Health Organisation (WHO) recommendation of 6 months. Early initiation was strongly influenced by breast feeding practices alongside the extent to which parents believed that their usual milk; that is, breastmilk or formula was fulfilling their infants' nutritional needs. The composition of diet and parents' approach to complementary feeding was closely aligned to traditional cultural practices; however, some contradictions were noted. The findings also acknowledge the pertinent role of the father in influencing the dietary practices of the wider household. Learning about both the common and unique cultural feeding attitudes and practices held by parents may help us to tailor healthy complementary feeding advice in the context of increasing diversity in the United Kingdom.

**Key messages**

- Irrespective of ethnicity, the majority of parents introduced solid foods earlier than the recommended guidelines of 6 months, with South Asian parents before 4 months of age. Conflicting advice perceived infant readiness and cultural and familial influences influenced parents' decisions.
- Complementary feeding was viewed as an important transition that enabled the infant to adopt the parent's cultural diet. Approaches to complementary feeding and types of food offered were entrenched in familial and cultural traditions, with strong intergenerational influence.
- Future interventions should provide more timely advice (around 1–3 months) on the signs of readiness and the risks associated with early initiation of solids, incorporate culturally tailored nutritional advice and acknowledge parents existing socio‐cultural context and the influential roles of others.

## 1 INTRODUCTION

The complementary feeding period represents an important developmental phase, with a transition from a child's dependency to independence (Alberts, 1994). The introduction of solid food plays an important role in the development of eating behaviour and oral‐motor skills including chewing, swallowing and speaking (Stevenson & Allaire, 1991), supporting the development of healthy and enjoyable eating habits (Foote & Marriott, 2003). Current guidelines recommend exclusive breastfeeding for the first 6 months, introducing complementary foods or drink from 6 months onwards (NHS, 2015; World Health Organization, 2002). Adherence to these guidelines is important as early initiation of complementary feeding (at 4–6 months of age) is associated with increased risk of multiple adverse health outcomes including childhood obesity (Wang et al., 2016), allergies (Burr, 1983), childhood respiratory illness (Wilson et al., 1998), iron deficiency and anaemia (Oski & Landaw, 1980). Despite this, and although knowledge of the complementary feeding guidelines is reportedly high (Department of Health, 2010), a large proportion of parents in the United Kingdom initiate complementary feeding before 6 months old (Bolling, Grant, Hamlyn, & Thornton, 2007; NHS Digital, 2010). The early introduction of solid foods has been linked to a range of different and often interrelated factors such as younger maternal age, low maternal education, low socio‐economic status and absent or reduced duration of breastfeeding (Wijndaele, Lakshman, Landsbaugh, Ong, & Ogilvie, 2009). However, the factors underlying these associations are complex (Pearson, Hunter, Cook, Ialongo, & Kellam, 1997), and we require a better understanding of why parents do not adhere to these recommendations.

The types of food offered to infants during complementary feeding is also important (Public Health England, 2017; World Health Organization, 2020). Current U.K. guidelines suggest that first foods should include a wide range of foods and flavours, beginning with fresh vegetables and soft fruits, progressing onto sources of protein, starchy foods and full fat dairy products (Foote & Marriott, 2003; NHS Choices, 2018). Allergenic foods, such as peanuts, cows' milk and shellfish, are advised to be carefully introduced into the infant's diet in small amounts and one at a time (Public Health England, 2017), with certain foods avoided during the complementary feeding period (e.g., those high in salt and sugar, honey, soft unpasteurised cheese, raw shellfish and fish high in mercury) (NHS, 2019). Commercial baby food is also considered less nutrient dense compared with home cooked foods with a much higher sugar content (García, Raza, Parrett, & Wright, 2013). However, qualitative research has suggested that parents are less clear about these feeding guidelines, feeling uncertain about what to feed their infants and how to engage in healthy complementary feeding (Harrison, Brodribb, & Hepworth, 2017). Research also suggests that there are cultural variations in the types of foods introduced by parents. For example, Pakistani, Bangladeshi and Indian mothers may have a preference for introducing sweet early foods such as egg custard and sweet rice with higher usage of commercial baby foods that they perceive as high quality (Ethnic Dimension for COI and DH, 2008). Whereas among Black African and Caribbean communities, there has been a tendency for introducing more savoury foods with less reliance on commercial baby food (Ethnic Dimension for COI and DH, 2008). Therefore, while knowledge is growing regarding the choice of foods offered by mothers from different cultural backgrounds, less is known about what influences these choices and if this differs across cultural groups. In addition, despite a recent rise in migration from Eastern European countries (ONS, 2020), there is still limited research evidence and knowledge of the complementary feeding practices used among parents from these areas (Townsend & Pitchford, 2012).

Traditional approaches to complementary feeding have focused on spoon‐feeding, beginning with smoothly blended foods, progressing to textured foods with an infant progressing to family based finger foods as they grow (World Health Organization, 2009). However, the growth in popularity of an alternative approach, ‘baby‐led weaning’ (BLW) (Brown, Jones, & Rowan, 2017), has led to a rise in research exploring the impact of different complementary feeding methods on food preferences, BMI and health‐related outcomes (Brown, Jones, & Rowan, 2017; Taylor et al., 2017). Within the BLW approach, instead of blending foods, infants are allowed to self‐feed family foods from 6 months of age, with an emphasis on allowing the infant to choose what they eat, how much they eat and to be part of family meal times (Rapley & Murkett, 2008). A number of possible benefits of BLW have been proposed including lower maternal anxiety and control around feeding (Brown & Lee, 2011a, 2011b), the development of healthier food preferences and reduced food fussiness in early childhood (Taylor et al., 2017; Townsend & Pitchford, 2012), enhanced motor skills (Rapley & Murkett, 2008) and a lower risk of obesity possibly due to more effective self‐regulation of energy intake (Brown & Lee, 2011b, 2015; Rapley & Murkett, 2008). However, risk of choking and concerns over failure to thrive (from both parents and health care professionals have also been noted within the literature (Cameron, Heath, & Taylor, 2012), and there is a lack of strong evidence to determine its true effectiveness (D'Auria et al., 2018; Taylor et al., 2017). Importantly, BLW is more common among higher socio‐economic groups (Brown & Lee, 2011a), and this can often confound findings (Taylor et al., 2017); further research among more diverse communities is essential. While current recommendations do not necessarily advocate one approach over another, the NHS does suggest that finger foods should form an important role in complementary feeding in helping infants develop co‐ordination and autonomy (NHS, 2019). Despite the lack of rigorous scientific evidence, the feasibility and safety of BLW approach have been recognised (Fangupo et al., 2016; Taylor et al., 2017), and the popularity of BLW is rising (Brown, Jones, & Rowan, 2017). As such, it is important that research continues to explore the different approaches to complementary feeding used by parents.

Overall, evidence suggests that complementary feeding practices and adherence to health recommendations are influenced by a range of different and often interrelating factors such as socio‐economic and cultural factors. However, the factors underlying these associations are complex, and we require a better understanding of what underpins and influences parents' knowledge, beliefs and practices relating to the initiation and continuation of complementary feeding. Given that the Black, Asian and Minority Ethnic (BAME) population represent 14% of the total U.K. population (ONS, 2018), there is limited awareness of how complementary feeding approaches vary across diverse populations with studies often limited to high socio‐economic groups. Further, despite fathers expanding role in child rearing (Khandpur, Blaine, Fisher, & Davison, 2014; Sarkadi, Kristiansson, Oberklaid, & Bremberg, 2008), they are often underrepresented in child nutrition research (Khandpur, Blaine, Fisher, & Davison, 2014). In order to develop and implement effective complementary feeding interventions, it is essential to better understand the knowledge and experience of both mothers and fathers from broad socio‐demographic groups and how these influence their behaviours.

Drawing on the views of mothers and fathers who reside in an ethnically diverse and deprived community, this study aimed to examine parents' knowledge, beliefs and practices of complementary feeding. In particular, it explores the initiation of complementary feeding, the types of foods offered during this phase and parents' approach to complementary feeding and the factors that inform and underpin parental knowledge, beliefs and practices relating to this.

## 2 METHOD

### 2.1 Setting

The study site for this study was Luton, UK. Luton is an ethnically diverse town with a population of just under 220,000 and is only one of three towns in the United Kingdom to have a White British population of less than 50%. Luton has a large South Asian (Pakistani, Bangladeshi and Indian) population, mainly Pakistani and Bangladeshi (21.1%), a significant African‐Caribbean population, and has seen a recent increase in migration from EU countries, most commonly Poland (UK Populations, 2019). Luton also experiences high rates of deprivation when compared with other parts of the United Kingdom (Research and Geospatial Information Team, 2011), with large sections of the visible BAME community residing in the most deprived wards.

This study focused on the five most deprived wards in Luton (Research and Geospatial Information Team, 2011). Table 1 presents a breakdown of the socio‐demographic characteristics of these wards. These wards have higher levels of obesity prevalence, lower fruit and vegetable consumption and a higher than average chance for children becoming overweight or obese when compared to average of Luton and England (Health Profile, 2012).

**TABLE 1:** Socio‐demographic characteristics breakdown across the five most deprived wards in Luton

WARD	Socio‐demographic characteristics
**1**	The local population is younger than Luton as a whole. Higher proportion than the Luton average of non‐White ethnic groups with the majority being of Pakistani and Bangladeshi ethnic origin.
**2**	This ward has a higher proportion of non‐White ethnic groups, in particular, Pakistani and Bangladeshi. The majority of residents within this ward describe themselves as practicing Muslim (Islam).
**3**	This ward has a higher percentage of lone parent families with dependent children when compared with the Luton average. This ward has a predominantly White population with a recent high influx of Polish immigrants.
**4**	This ward has a higher proportion of Black African, Caribbean and White British residents compared to the rest of Luton. This ward is predominantly Christian or non‐religious.
**5**	This ward has a higher percentage of White Europeans (e.g., Polish), Black African compared with the rest of Luton. There is a high student population in this area and a distinctly higher proportion of the population residing in purpose‐built blocks of flats.

### 2.2 Participants and methods

Focus groups were used, as they are a particularly valuable resource to help identify aspects of health behaviour related to healthy diet and weight to develop culturally sensitive interventions (Culley, Hudson, & Rapport, 2007; Krueger, 1994; Powell & Single, 1996). A purposive stratified sampling strategy was used to recruit focus group participants across the five most deprived wards in Luton, UK (Table 2).

**TABLE 2:** Sample of those included in study

Focus group	Ward	Ethnicity	Gender	Age	*N*
1	1	Pakistani	Female	31–45	8
2	1	Bangladeshi	Female	21–30	5
3	1	Pakistani	Male	31–45	5
4	1	Bangladeshi	Male	21–30	6
5	2	Pakistani	Female	21–30	5
6	2	Pakistani	Male	21–30	4
7	2	Bangladeshi	Male	31–45	4
8	2	Bangladeshi	Female	31–45	5
9	3	White British	Female	21–30	4
10	3	White British	Female	31–45	4
11	3	White British	Male	21–30	7
12	3	Polish	Male	21–30	3
13	3	Polish	Male	31–45	5
14	4	Black African	Female	31–45	2
15	4	Black African	Female	21–30	8
16	4	Black Caribbean	Male	21–30	5
17	4	Black Caribbean	Male	31–45	4
18	5	Black African	Female	31–45	2
19	5	Black African	Female	31–45	3
20	5	Polish	Female	26–37	8
21	5	Polish	Female	33–39	6
22	5	Polish	Female	27–35	3
23	5	Polish	Male	21–30	2
24	5	Polish	Male	31–45	2

The inclusion criteria were mothers or fathers aged between 21 and 45 years old with at least one child aged between 0 and 5 years old. Parents were required to reside in one of the five most deprived wards in Luton and to self‐identify as being from the White British, Pakistani, Bangladeshi, Black African/Caribbean or Polish ethnic groups (using the ethnic group categories from the Census (2011) (ONS, 2011). Participants who did not speak English were included in the study to ensure that we captured the views of a range of parents regardless of their level of English language fluency.

All focus groups were stratified by self‐identified ethnicity, age (21–30 and 31–45) and gender. On a technical level, gender segmentation provides a useful approach to enable groups to talk more openly and facilitate discussions of common experience (Morgan, 1995), which is integral when engaging with diverse and hard to reach communities (Randhawa & Darr, 2001; Sharp & Randhawa, 2017). This is also particularly important given that some cultural groups we interviewed (e.g., South Asian) still may hold gender segregation practices that may have impacted on open participation (Ali, McLean, & Rehman, 2012; Morrison‐Beedy, Côté‐Arsenault, & Feinstein, 2001). On a theoretical level, segmented focus groups by age and gender enabled the researchers to make cross‐group comparisons, allowing similarities and differences across groups to be explored to ascertain contrasting viewpoints (Halcomb, Gholizadeh, DiGiacomo, Phillips, & Davidson, 2007; Morgan, 1995)**.** Table 2 presents the demographic composition of all focus groups. There were 24 single sex focus groups based on self‐identified ethnicity, with a total sample of 110 parents (63 mothers and 47 fathers). Participants' ages ranged between 21 and 45 years across the five defined ethnic groups: White British (*N* = 15), Pakistani (*N* = 22), Bangladeshi (*N* = 20), Black (*N* = 24) and Polish (*N* = 29).

Focus group discussions were conducted between the period of February 2014 and January 2015. All focus group discussions took place at a time and place convenient to participants with many of the focus groups taking place within local community centres. Each focus group session lasted approximately 90 min and was audiotape‐recorded with participants' permission. All focus groups were conducted by bilingual, gender‐matched facilitators. This approach enabled all non‐English speaking participants to take part and encouraged rapport building with the facilitator, allowing for more insightful discussions. All facilitators had extensive experience of interviewing and conducting focus groups within community settings and were not known to any of the participants who took part in this study. At the end of the focus groups, all participants were given a £10 high street voucher as a good will gesture for their participation in the focus group. These incentives were provided to overcome potential costs, for example, travel costs, to the participants to taking part in the focus group.

The topic guide was developed collaboratively as part of the multi‐disciplinary research group who have expertise in areas surrounding: eating behaviour, nutrition and health inequalities. Open‐ended questions explored parent's knowledge surrounding complementary feeding and beliefs and practices of the complementary feeding period. This focused upon when parents initiated complementary feeding including the type of foods offered, parents' approach to complementary feeding alongside the factors that influenced these attitudes and behaviours. This study is a smaller substudy of a larger research programme that explored parents' views on healthy diet and weight for children aged 0–5 (findings from the rest of the project are not presented here).

### 2.3 Data analysis

Focus groups were translated (where necessary), transcribed and analysed using framework analysis (Ritchie & Lewis, 2003). Coding was applied manually to all narrative, which varied from small sections of data (parts of sentences) to whole paragraphs. F. P., E. C. and C. P‐J. then independently coded three transcripts and then discussed the labels assigned to each passage. All themes and subthemes were discussed with the authors and agreed upon after discussion. An analytic framework agreed with the research team was developed with a brief description of all codes and applied to all transcripts. The final stage involved summarising and synthesising the range and diversity of coded data by refining initial themes and categories. Abstract concepts were developed collaboratively through the identification of key dimensions of the synthesised data and making associations between themes and concepts. NVIVO v11 was used to manage the data that allowed for ease of data retrieval and a high level of working familiarity with the data under consideration. Core findings were also discussed with the interviewer to determine that they were an accurate reflection of the interviews with no inaccuracies found.

### 2.4 Ethical considerations

This study was conducted according to the guidelines laid down in the Declaration of Helsinki and all procedures involving research study participants were approved by the University of Bedfordshire Institute for Health Research ethics committee (IHRREC367). Written informed consent was obtained from all subjects.

## 3 RESULTS

Three main themes emerged, which included (1) initiation of complementary feeding, (2) diet (consistency and composition) and (3) approaches to complementary feeding. These themes and their resulting subthemes are discussed in detail below.

### 3.1 Theme 1: Initiation of complementary feeding

Parental initiation of complementary feeding was earlier than the recommended 6 months across the majority of parents. Pakistani, Bangladeshi and Polish parents introduced solid foods the earliest (around 3–5 months), with White British parents around 4–5 months. Black African and Caribbean parents were the only parents to wean their infant at the recommended 6 months. The decision of when to initiate complementary feeding was influenced by a number of factors outlined within the themes as follows.

#### 3.1.1 Professional advice versus personal preferences

The majority of mothers irrespective of ethnicity stated that they were given advice by a health care professional, most commonly a health visitor that their infant should not be given complementary foods until 6 months. Most parents admitted that they disregarded this advice with only a few parents mentioning the potential problems associated with early initiation of complementary feeding (e.g., infants' stomach is not fully developed). The predominant reason for being non‐compliant was that this advice conflicted with their own personal views (based on intuition regarding infant readiness) and values imbedded within their familial and cultural context or previous recommended advice. The change in guidance (from 4 to 6 months) of when to initiate complementary feeding was regularly discussed with a lack of understanding of why the changes were made. This was most notable among White British mothers: ‘you know you have to wait until six months to start feeding them but erm previous generations of kids it was four months so I was kind of like oh I don't know if I should wait’ (White British mother, aged 21–30). However, on occasions where an infant had a medical concern, for example, born premature, parents were more likely to adhere to professional advice.

#### 3.1.2 Perceived infant readiness

The decision of when to introduce complementary foods was based on the extent to which parents believed that milk (breast/artificial) was fulfilling their infants nutritional needs (breastfeeding practices). The notable longevity of breastfeeding among Black African mothers compared with other ethnic groups alongside their positive beliefs regarding the nutritional value of breast milk both were influential in their decision to not introduce complementary foods until the recommended 6 months: ‘you don't see sickness in breastfed children they are healthy and strong, breastmilk is all they need like a cow gives their milk to calf’ (Black African mother, aged 31–45). For South Asian and Polish parents, the decision to introduce solid/semi solid or soft foods also coincided with the mothers' decisions to stop exclusively breastfeeding (earlier than 6 months) where it was felt that a different source (e.g., complementary foods) would be needed to provide the infant the necessary nutrients he or she needs. This was particularly common among South Asian mothers who disclosed concerns of their infants' low birth weights with concerns of failure to thrive.

Knowing when their infant was ready for solids was based on readiness cues alongside mother's intuition. Readiness cues included putting fingers in mouth, chewing, showing an active interest in food and others eating: ‘They said don't feed him until six months, but I felt he was really hungry, but she strongly said six months that's when you start feeding him. But I can tell he was hungry he was interested when everyone having their dinner, chewing, he was hungry’ (Bangladeshi mother, aged 21–30). Some mothers, particularly among Pakistani and Bangladeshi focus groups felt mother's intuition is the most important regarding decisions of when an infant is ready to eat solids.

So, these 20 years old midwives are going to come and tell me what to do, I am not going to listen to them. I respect them but I am sorry I am going to give my child [solid foods] at 4 months. I am not going to give them [solid foods] at 6 months.

(Pakistani mother, aged 31–45)

Cultural and familial influence

Traditional cultural practices and familial influences were particularly pertinent to the initiation of complementary feeding. For some parents, the initiation of complementary foods was closely aligned to traditional practices from their country of origin. Black African mothers, for example, revealed that culturally, they do not initiate complementary feeding until they are 6 months, as it is not until then that infants are perceived as ready to swallow African foods: ‘I think even culturally 6 months; because where I come from, from Nigeria, you start weaning – although with African food, like with okra, we give that to them so that they can swallow’ (Black Mother, aged 21–30). This was also true of Polish parents who felt that the initiation of complementary foods should be much earlier (4–6 months) as would be common practice in Poland.

However, South Asian (Bangladeshi and Pakistani) parents appeared to introduce solid foods much earlier than would be expected traditionally in their native country, where delays in introducing solid foods are common. As one father states, ‘you slowly introduce solids like even at the age 1 or 2 months, 2 months if you feed them right, but again it's in our culture isn't it, because rice is there and then you feed them that’ (Bangladeshi Father, aged 31–45). Many South Asian parents felt pressure to get their infant adjusted to solid foods as soon as possible, which could present some challenges. For example, one Bangladeshi father discusses the challenges of getting his son to eat more ‘solid’ foods: ‘he started eating things like solid food here and there. The reason why I had to try to, I had to force him to feed, because we would try to set up regular meals for him, that's what it was. He was eating beforehand, but milk was the regular feed and that was just snacks. Then what we wanted to do now was to shift to the food being the regular meal and maybe just the milk being the gap filler in that sense.’ (aged 31–45).

Family represented a strong influence on the initiation of complementary feeding across all parents. The mother and/or mother‐in‐law (among Pakistani and Bangladeshi mothers) were the most influential and would often tell their daughter (in law) when their child was ready to eat solid foods based on both their observations and past experiences of complementary feeding. Some of the South Asian mothers who lived with their family (or in laws) were extremely influenced by their extended family on when and how to initiate complementary feeding. As one mother states, ‘I live with my mother in law so obviously she gave me loads of tips and stuff, she said when they're about three four months, put baby rice in the milk and apparently that keeps them, that starts off the weaning process kind of thing’ (Bangladeshi Mother, aged 31–45). South Asian mothers also viewed their sister(s) (and sister(s)‐in‐law) who were already mothers as playing an important role in the initiation of complementary feeding. This would be through observations of what they had seen their sister (in law) do and in result of discussions surrounding complementary feeding with them. Grandparents, ‘elders’, were particularly influential among Black African parents. They held an important ‘teaching’ role, passing on their knowledge and advice of what had worked well for them and their views were widely respected by the parents particularly the fathers.

Our regime sounds similar too [to other participant], where he said Grandparent's influenced a lot. It was my grandparents in this case, they sent up a lot of food for my sons as babies. Things like Cream of Wheat, Farina porridge, etc.

(Black Father, aged 21–30)

### 3.2 Theme 2: Complementary foods: consistency and composition

There were a range of factors which related to complementary feeding practices among the parents which are outlined within the themes below:

#### 3.2.1 Cultural diet, beliefs and practices

Complementary feeding was viewed among all focus groups as the transition from milk to the family's cultural diet. The introduction of solid foods across all parents centred around the slow introduction of softer foods most commonly baby rice, fruits and vegetables, adapted across all parents to reflect ethnically sourced foods and their cultural diet (see Table 3). One African mother revealed that her child did not have any ‘English’ 1 food until they were 1 year old, ‘he went for the cultural food and it was mashed banana and plantain, my boys didn't have anything English until his first birthday party’ (Black African Mother, aged 21–30). Polish parents introduced their infant on softer foods such as rice gruel and millet groats and soups as their infant adapted to their family's diet. South Asian parents also offered pureed foods although these included more sweeter options including pureed fruit, custard and rice pudding.

**TABLE 3:** Age of initiation, approach and examples of first complementary foods given to infants

	Initiation age	First foods	Progression foods	Approach to complementary foods
Pakistani/Bangladeshi	3–5 months	Boiled vegetables, fruit, semolina, Gutti (honey), rice pudding, homemade custard and khichri (dish containing rice and lentils)	Roti (flatbread made with wholemeal wheat flour), rice, Salan (thin gravy made from ingredients such as peanuts, sesame seeds and coconut), pumpkin dahl flavoured with spice (thick South Asian stew made from legumes such as lentils with various seasoning) and oils (olive oil and cheese olive oil), addition of salt and spices (e.g., turmeric and paprika)	Pureed
African/Caribbean	5–6 months	Boiled vegetables (celeriac, carrots, potatoes, plantain, pumpkin, sweet potato, yam and okra), baby rice and gruel	Porridge (Farina and cream of wheat), semolina mashed bananas with butter, plantain banana, cornmeal and vegetable curries	Pureed
White British	4–5 months	Finger foods: chips, pasta, fruit and vegetables (soft boiled carrot, avocado and potato)	Family‐based meals	Baby led/combination
Polish	3–5 months	Millet groats, rice gruel (porridge), soups and fruit yoghurt	Adapted family‐based meals (to limit salt, spices and meats with additional vegetables)	Combination

Cultural beliefs regarding food were important determinants of diet composition among South Asian parents. Pakistani fathers for example, discussed the concept of ‘warm and hot’ and ‘cold’ foods in Asian culture where parents of the fathers (first generation migrants) believed that ‘warm and hot’ foods bring warmth while ‘cold’ foods cool down the body. With a colder climate in the United Kingdom, giving an infant colder food (e.g., milk and yoghurt) in Autumn or Winter for example would be viewed culturally as ‘unhealthy’. There was also a perception that South Asians' immune systems are not as strong as non‐Asians with reinforced these culturally held beliefs ‘if you give them something cold, well it could be a cultural thing or it could be genetic or because we are from different weather but actually what happens, as soon as they have it, they would have a cough or chest infection. We are weaker, you see, our immune system is not very strong as probably the non‐Asian person’ (Pakistani males, 21–30). In addition, there were some culturally important *warm* foods, such as honey (referred to as Gutti; believed to have healing and warming properties), which despite medical and professional advice, parents chose to give to their infant.

As complementary feeding progressed many parents (South Asian, Polish and African/Caribbean) revealed that they fed their infant foods straight from the family ‘pot’, an important aspect of adjusting the infant to their cultural diet. It became increasingly apparent that the role of the father across all focus groups appeared to play a very important role in influencing the dietary practices of the whole household and subsequently the foods the infant would be offered. The differences however emerged focused on the awareness and the motivation to adapt the household diet. Polish fathers were the most aware and receptive and frequently discussed the importance of adapting the whole family's diet to ensure that the family foods met the nutritional needs of the infant (e.g., limiting salt, spices and ‘heavy foods’, for example, processed meat and increasing vegetables). South Asian fathers who were viewed as the head of the household were more focused on getting the infant used to their diet so while they would process family foods (e.g., mashing) to make it more suitable, they would progressively add spices (such as turmeric and paprika) to the foods so that their infant would slowly get used to eating spicy food.

#### 3.2.2 Commercial versus homemade food

There was much discussion among parents regarding the use of commercial baby foods. There was an acknowledgement among all parents that homemade foods were nutritionally healthier than commercial baby foods, with the value of organic foods particularly important among Black fathers. There were also added advantages disclosed among some mothers regarding homemade foods including the ability to add calories and add meat (Bangladeshi mothers). South Asian (Pakistani and Bangladeshi) fathers were particularly positive towards convenience‐based baby food with many suggesting that their infants preferred it to home‐made food, which could have been related to the infant being unfamiliar to textured foods being given, for example, mashed potato, porridge and Khirchri (rice and lentils).

Polish fathers held the most negative views towards the use of commercial foods. They acknowledged that homemade food took additional time and effort; however, for them, this was outweighed by the health benefits for the child. However, the focus groups revealed some contradiction with commercial baby food still commonly used among Polish parents although mostly during the early stages to help the infant transition from breast to solid foods: ‘we had a challenge after weaning away from the breast so we started to feed him food from little jars’ (Polish father, aged 21–35). Following this food was homemade, often with the fathers input and while this took much more effort, it was considered worthwhile: ‘And now we started to basically cook on our own, we made ourselves some groats, we'd make them with some vegetables. We also invested in a blender and we basically made things ourselves. Most things. So, hmm, let's say that it required a lot of input and effort, but it was worth it’ (Polish father, 31–45).

White British and South Asian (Pakistani and Bangladeshi) fathers viewed commercial food as a convenient substitute to preparing homemade food: ‘It's easier, more convenient, just warm them up. You also get a lot of flavours. Blending just takes so much time, tinned stuff is more convenient but natural stuff is better, I guess it depends on how much time you have got’ (Bangladeshi father, aged 31–45). South Asian fathers viewed commercial baby food as a more permanent alternative through complementary feeding while the infant gets used to eating the families' cultural diet: ‘to be honest about baby foods I'll say that from 5 months to about 10 11 months not even that then you buy products from your local say for example Heinz baby food but then after that it's just more or less eating our own food’ (Bangladeshi father, 31–45). White British fathers also viewed commercial ready‐made baby food as an easier and often more convenient alternative to fresh food; however, they appeared to have less knowledge about what food is considered ‘healthy’ for babies. This may be a result of complementary feeding being viewed as the mother's role.

#### 3.2.3 Influence of family and friends

Across all focus groups, family were identified as an important influence on parents' complementary feeding practices, though the strength and value that this advice held varied slightly. The influence came from both their experience of their upbringing as well as advice that they had received. Pakistani, Bangladeshi and Black parents were particularly influenced by respected members of their family and the wider community. These influences were entrenched through cultural practices by elders and other esteemed family members.

Friends who had children were also particularly influential among White British and Bangladeshi mothers. One white British mother states: ‘I think friends who have children also have a big influence on how you raise yours as well’ (aged 21–30). However, for Polish, Asian and Black African/Caribbean parents, elders (in particular, the mother and mother‐in‐law) were seen as the most influential: ‘The In‐laws, that is nevertheless the biggest treasure [of knowledge] and it cannot be avoided, especially amongst Poles, where I believe In‐Laws, i.e. the grandmothers, have the most to say, they are the mothers who raised children.’ (Polish Father, 31–45). The sister (and sister‐in‐law) was also influential among South Asian mothers. Mothers felt that they provided useful practical advice, which included how to prepare purees, how to cook and what foods to provide. The sister often had children at a similar age to the parent; therefore, they could draw on personal observations of how they have weaned their children and follow suit. These family members also influenced the use of commercial baby foods among South Asian parents: ‘I haven't started weaning yet but when I do I'm thinking my sister, what she does, I saw her starting with baby rice and then you can move on to jar food’ (Bangladeshi mothers, 21–30).

Most parents held a positive view of the eating practices followed by the older generations. However, some parents, mostly South Asian and White British younger parents, were more critical of their parents' diet, suggesting that they had outdated views on what constitutes a healthy diet, for example, a diet high in oil and salt and low consumption of vegetables (South Asian) and a diet high in fat and sugar and reliant on processed foods (White British). Despite this, some parents felt huge pressure by their elders to provide their children with the culturally traditional foods that they were brought up on regardless of if they felt they were healthy or not, often leading to confrontation ‘It stems from the pressure of our parents, if you try to say “We want to try to give our child XYZ” or we want to try and give them some healthy options, your father will say “what's wrong with the dal and Roti that we've been eating for the last 30/40 years? Nothing happened to us’ (Pakistani Father, 31–45). Similarly, a White British mother disclosed that her parents told her: ‘just add a bit of gravy to it, if it's really dry, but apparently oh you're not allowed to give kids gravy it's too salty for them, so I, well I told them and she said they are being ridiculous I still did anyway’ (White British Mothers, 21–30). Evidently, families faced an overwhelming pressure to follow parental advice and challenging such advice was often problematic.

### 3.3 Theme 3: Approach to complementary feeding

#### 3.3.1 Spoon‐fed versus baby‐led weaning

South Asian (Pakistani and Bangladeshi) and Black (African and Caribbean) parents adopted spoon‐fed approaches to complementary feeding, starting with thin gruels (similar consistency to milk), which were then progressed to more thicker purees with solid ‘whole’ foods not introduced until much later (normally 10/11 months). There was limited discussion across these focus groups regarding finger foods with the exception of a few parents who offered their infant ‘baby biscuits’, for example, Rusks. Some of these mothers revealed that they were told by a health care professional to offer finger foods alongside the pureed foods during health visitor checks; however, this was something they were familiar with and was not traditional practice: ‘We just give it by the spoon and feed the baby, I did not understand about that [finger foods], it is not something that we do’ (Pakistani Mother, 31–45).

In contrast, White British and Polish parents were stronger advocates of ‘baby‐led weaning’ (letting the baby feed themselves), although it was often done in conjunction with pureed foods (combination feeding). Mothers felt that baby‐led weaning enabled their infant to learn how to chew, rather than just swallow (as commonly used in pureed foods). White British mothers started baby‐led approaches from the onset, providing their child with small bits of food including chips, pasta, fruit and vegetables. However, Polish parents stated that they introduced pureed fruits and vegetables to start and then progressed their infants to solid foods to allow infants the opportunity to hold food, eating with some independence. Advantages of this approach focused on how it enabled their infant to explore different flavours and textures of foods and provided their infant more control over what they were eating and how much, ‘She [child] sometimes squishes her food, plays with it but that is what it is all about, learning about different foods and their textures’ (White mother, aged 31–45). White mothers also felt that offering finger foods was a much cheaper alternative. Family mealtimes particularly among Polish parents were viewed as an important opportunity for letting their infant explore ‘family’ foods. Polish fathers, for example, discussed allowing their infant to take a lead in initiating eating, for example, letting them observe them eat and preparing them smaller portions of food from their plate for their infant to explore. Nonetheless, the possibility of choking was a notable concern and barrier, with some mothers reporting that had experienced an event of choking when eating solid food, normally during the early stages. There was also a fear that they were not getting enough nutrition most commonly expressed among White British mothers who were exclusively baby‐led weaning.

## 4 DISCUSSION

The findings from this study provide a current qualitative exploration of the knowledge, beliefs and practices surrounding complementary feeding among both mothers and fathers who reside in a culturally diverse and deprived community. Specifically, discussions were focused on the initiation of complementary feeding, the types of foods offered by parents during this phase and parents' approach to complementary feeding that sought to provide a valuable insight into the factors that inform and underpin parental knowledge, beliefs and practices relating to this. The findings revealed three main themes: (1) initiation of complementary feeding, (2) diet (consistency and composition) and (3) approaches to complementary feeding which are discussed in detail below.

### 4.1 Initiation of complementary feeding

Despite good knowledge of the recommended guidelines, with the exception of Black African and Caribbean parents, most parents introduced their infant to complementary foods earlier than the recommended 6 months, with some as young as 2 months. This supports previous evidence that early initiation of complementary feeding is commonplace within the United Kingdom (Bolling, Grant, Hamlyn, & Thornton, 2007) and suggests that within deprived communities, lack of adherence to guidance is common across most ethnic groups. Interestingly, the initiation of complementary feeding among Polish, Black African and Caribbean parents appeared to reflect traditional approaches found within their country of origin. However, South Asian parents revealed that they introduced complementary foods notably earlier than found among traditional practices in their country of origin. South Asia, for example, is shown to have the lowest rates of early introduction of complementary foods worldwide (Hazir et al., 2012; White, Bégin, Kumapley, Murray, & Krasevec, 2017) with delayed introduction found higher in lower income households and among mothers with lower education status (Hazir et al., 2012; Manikam et al., 2017; A. Patel et al., 2012). Therefore, this could represent a shift of attitudes regarding infant feeding practices to those more closely aligned within the United Kingdom. These findings also highlight that conversations with parents, particularly those from South Asian communities, regarding the initiation of complementary feeding, need to be taking place earlier (1–3 months).

Parents discussed different influences on their decision about when to give solid foods, namely, conflicting advice, perceived infant readiness and cultural and familial influences. Points raised regarding confusion over the rationale for changing guidance from 4 to 6 months shows that while parents are aware of the 6‐month recommendation, they have limited understanding as *why* these recommendations have changed and *why* it is important to wait until 6 months rather than 4. No parents discussed the multiple adverse health outcomes associated with early initiation of complementary feeding (NHS Digital, 2010; Oski & Landaw, 1980; Wang et al., 2016; Wilson et al., 1998). This suggests parents may need more information regarding the rationale and evidence underpinning these guidelines to increase parents' trust and to improve adherence.

The most common reason for early initiation of complementary feeding that related to perceived infant readiness was the concern that their infant was hungry and was not getting enough nutrition through their usual milk. This also coincided with breastfeeding practices; for example, parents who breastfed exclusively for at least 6 months (e.g., Black African and Caribbean mothers) were more likely to believe that breast milk was fulfilling their infants' nutritional needs and therefore more likely to introduce complementary foods at 6 months, whereby early breastfeeding cessation among South Asian, White British and Polish mothers was closely related to introduction of complementary foods. This is further supported by research conducted in Poland that found that women who do not exclusively breastfeed during the early months, alongside those who receive infant formula following hospital discharge are more likely to initiate complementary foods earlier than those who exclusively breastfeed (Schiess et al., 2010; Zielinska, Rust, Masztalerz‐Kozubek, Bichler, & Hamułka, 2019). Early initiation of complementary feeding could therefore be related to the notable reduction in exclusive breastfeeding found within the BAME community (Choudhry & Wallace, 2012; Kelly, Watt, & Nazroo, 2006). As such, a more concerted effort is needed to develop and implement effective breastfeeding interventions among culturally diverse communities to motivate and support mothers to exclusively breastfeed where possible for the infants' first 6 months of life.

It has been suggested that the rigidity in complementary feeding guidelines, based on generic age, can create conflict when mothers are also trying to respond to developmental signals (Arden, 2010; Hetherington, 2011), a perception reflected by the parents within this sample. While consistency in messages from health care professionals being closely aligned to U.K. guidelines is important, given reports that only 1% of parents adhere to 6‐month guidelines (Bolling, Grant, Hamlyn, & Thornton, 2007), this must be delivered in a way that acknowledges parents existing attitudes and beliefs and empowers them to make their own, well‐informed choices. In addition, more consideration should be given to the cultural and familial influences that impact on adherence to guidelines. Among BAME groups, the parent's decision on when to initiate complementary foods was embedded within traditional cultural values. Family members (namely, grandparents and siblings) were influential across all groups, but this was most notable within multigenerational households. While multigenerational households can be beneficial for support (Pearson, Hunter, Cook, Ialongo, & Kellam, 1997), previous research has suggested that strong influence from a maternal grandmother (Alder et al., 2004) or using them as a principal source of infant feeding advice (Tarrant, Younger, Sheridan‐Pereira, White, & Kearney, 2010) is associated with an increased risk of starting complementary food before 12 weeks. Similarly, associations can be inferred from this study, particularly among South Asian families.

### 4.2 Diet (consistency and composition)

The composition of introductory foods discussed was reflective of those traditionally given in the parent's country of origin. For example, Black (African and Caribbean) parents introduced gruel, normally derivative from maize meal traditionally given spoon fed to the infant (Faber, Laubscher, & Berti, 2016). South Asian parents introduced cereals and khichri (preparation of rice with lentils), which also represents a traditional diet for introductory foods (Manikam et al., 2017). However, South Asian and Polish parents were more likely to introduce and progress their infant using commercial baby foods, a far contrast to traditional cultural practices. Previous research compared feeding practices among Pakistani‐born mothers who reside in the United Kingdom to those who live in Pakistan, and similarly, it was found that those who resided in the United Kingdom were more likely to use convenience foods (Sarwar, 2002). This therefore may strengthen the argument surrounding acculturation and feeding practices among South Asian parents but also may suggest that more newly acculturated ethnic groups such as Polish may also be adapting their approaches to complementary feeding.

Many parents across the focus groups expressed the importance of their children eating their cultural diet. This was particularly pertinent in South Asian and Black African/Caribbean focus groups where traditional foods would be provided throughout the complementary feeding phase. The aim would then be for the child to adopt the parent's cultural diet, and complementary feeding was the process to facilitate this. However, very few (except Polish families) discussed the importance of adapting their diet to meet the nutritional needs of their infant, for example, limiting salt and spices and increasing fruit and vegetables. Parents are pivotal in children's feeding practices, from selecting the foods of the family diet to modelling eating practices (Savage, Fisher, & Birch, 2007). Importantly, feeding practices are potentially modifiable, and with relevant advice, parents could support the development of long‐term healthy culturally embedded eating patterns and behaviours in their children. Interventions should consider incorporating culturally tailored nutritional advice to inform parents about how and when cultural foods can be used in the complementary feeding process and provide advice on how family meals can be adapted to meet the growing nutritional needs of the infant.

The influence of the family was also pivotal for many of the parents when making decisions regarding complementary feeding. The mother (in law) was viewed as the most influential among the Polish, Asian and Black African/Caribbean focus groups. It has been suggested that the significant influence of cultural and familial beliefs passed down between generations can slow the uptake and adherence to contemporary recommendations, with more traditional feeding practices prevailing (Gildea, Sloan, & Stewart, 2009). Similarly, within this study, while the immediate family were mostly viewed as a positive influence, there were some parents who felt that their diet during their childhood was perhaps not as healthy as it should have been. Many of these parents felt that while they perhaps wanted to adapt their family's diet, they felt pressured by their elders to continue such traditions. It was also common that the family dietary practices and advice from grandparents were in direct contradiction to health care professional's advice, which would also create a challenge for parents. Community‐driven interventions should be centred on empowering parents in how to manage family expectations and cultural traditions and provide strategies on how to address difficult conversations within the family network. This research also highlights the importance of including the wider family as targets for messages about nutrition (Pak‐Gorstein, Haq, & Graham, 2009).

### 4.3 Approach to complementary feeding

There were differences noted between the ethnic groups relating to their approach to complementary feeding. White British and Polish parents most commonly used baby‐led weaning, whereas South Asian (Pakistani and Bangladeshi) and Black (African and Caribbean) relied on more parent‐led spoon‐feeding approaches. Previous research among Polish groups is rare, and although findings from one study suggests that Polish parents perceive many benefits to adopting a BLW approach, this is limited to a sample of high SES parents residing in Poland (Poniedziałek, Paszkowiak, & Rzymski, 2018). Similarly, BLW has been consistently associated with higher SES and education level in U.K. mothers (Brown & Lee, 2011b; Cameron, Heath, & Taylor, 2012). This study suggests that BLW may also be a growing approach among British and Polish groups from more deprived communities within the United Kingdom and provides a strong rationale to continue to explore this approach and its potential benefits among lower SES families.

Parents' approach to complementary feeding was closely aligned to cultural norms and parents' cultural beliefs. For example, research has suggested that spoon feeding particularly among South Asian families has an emotional connotation, viewed to signify a mothers' love for her baby (Department of Health, 2010). Other factors that influenced complementary feeding practices among South Asian and Black African/Caribbean parents included parental control of feeding, worries about choking and a lack of awareness of BLW approaches. White British and Polish parents felt that a BLW approach may fail to meet the nutritional needs of the infant. However, this is unsupported by recent RCT evidence that has suggested that BLW and spoon feeding do not differ in their impact on the development of an appropriate BMI (Taylor et al., 2017). Further, given that breastmilk or formula can continue to provide all that infants require in the way of macronutrients beyond 6 months (NHS Choices, 2018), parents require a better understanding that the early weeks of complementary feeding need not be concentrated on calorie intake, but rather focus on providing a ‘fun’ opportunity for their infant to explore a variety of textures and flavours as they learn to become an autonomous eater (Foote & Marriott, 2003). This was not discussed among the parents within this study other than the White British mothers.

Research exploring the impact of different complementary feeding methods on food preferences, BMI and health‐related outcomes is still in its infancy and not without inconsistency (Brown & Lee, 2011a, 2011b; Fangupo et al., 2016; Taylor et al., 2017; Townsend & Pitchford, 2012). While current recommendations do not necessarily advocate one approach over another, the NHS does suggest that finger foods should form an important role in complementary feeding in helping infants develop co‐ordination and autonomy (NHS, 2019). Given that BLW has been identified as one of the strongest predictor of complementary feeding at the recommended age (Moore, Milligan, & Goff, 2014), and its feasibility and safety as a feeding approach has been recognised (Fangupo et al., 2016; Taylor et al., 2017), changes in guidance to promote this approach or a combined method may be useful. Reassurance from health care professionals to dispel fears surrounding choking risk, failure to thrive and macronutrient deficiency would also be well targeted.

The findings also highlighted the important role of fathers in influencing the dietary practices of the wider household particularly across the South Asian sample. For example, South Asian fathers were shown to be the least aware and resistant to adapting the wider family diet to meet the nutritional needs of the infant. This is also reflected in the literature where female spouses who despite wanting to instil positive dietary change are often met with resistance from fathers who are often unwilling to sacrifice taste (Patel, Phillips‐Caesar, & Boutin‐Foster, 2012). Considerations should therefore be made when tailoring nutritional advice to acknowledge the influence of fathers and their pertinent role in decision making within the household. Fathers also appeared to have less knowledge about what food is considered ‘healthy’ for babies, possibly reflected in their perception of the ease and convenience of commercial readymade food.

#### 4.3.1 Strengths and limitations

The importance of increasing engagement with socially disadvantaged communities and achieving good representation in research is pivotal to ensure that targeted public health interventions reflect the wider ethnically diverse population. However, the challenges of engaging with socially disadvantaged groups are well documented (Bonevski et al., 2014). This study, through the employment of a tailored and flexible recruitment strategy, enabled us to tackle some of these challenges. The use of single sex focus groups matched by age and ethnicity alongside bilingual fieldworkers who were also purposefully matched enabled us to achieve excellent participation with commonly underrepresented and hard‐to‐reach groups across all of the deprived wards targeted. This has enabled a richer understanding of views and experiences reflective of the wider community, rather than being focused on smaller subsections of the community, a common limitation of previous research (Corbett, 2000; Sarwar, 2002). Furthermore, the research design allowed for the inclusion of critical dimensions of age, sex and ethnic variation among the most deprived wards of a culturally diverse community. We also sought to include Polish families and fathers' views who are often underrepresented in child nutrition research. Adopting this approach has enabled us to identify and reveal useful and important patterns of similarities and differences where they exist.

The focus of this study was to determine beliefs and practices surrounding complementary feeding among ethnically diverse mothers and fathers. While we were successful in recruiting parents from a range of ethnic backgrounds across the five most deprived wards, there were challenges in the recruitment of Black African fathers and Black Caribbean mothers. Therefore, our findings may not be ‘representative’ of all ethnic groups views that may limit transferability. In addition, the interrelation between factors such as SES, education and ethnicity, evident within our sample, can make it difficult to unpick the relative importance of each (Cameron, Heath, & Taylor, 2012). However, this study was not attempting to uncover ‘facts’ in a statistical sense but was instead focused on uncovering how parents within a cultural group may present different experiences and perceptions in relation to complementary feeding practices, and how these may (or may not) challenge their cultural expectations (Hammarberg, Kirkman, & de Lacey, 2016). As part of the inclusion criteria, participants were required to have at least one child aged between 0 and 5 years. There is the potential that it may have been some time since parents have initiated complementary feeding that may have led to the potential for recall bias. Nonetheless, the findings of this study offer further insight into the issues surrounding diversity, which can be applied to our understanding of other similar communities both nationally and internationally. The findings illustrate the importance of culturally tailoring advice surrounding complementary feeding, including acknowledging the fathers and the wider family and social networks that exist. This study hopes to go some way in supporting wider public health policy and strategies to better support parents through the complementary feeding phase to improve positive health and developmental outcomes in children (Burr, 1983; Wang et al., 2016; Wilson et al., 1998).

## 5 CONCLUSIONS

This study examined knowledge, beliefs and practices of complementary feeding among parents who reside in a culturally diverse and deprived community. The findings revealed that irrespective of  ethnicity, most parents introduced solid foods earlier than the  recommended 6 months, which may suggest that SES rather than ethnicity may be a more important factor in early initiation of complementary feeding. Common myths, a lack of awareness of the developmental signs for readiness alongside familial and cultural beliefs, were all influential factors of early initiation. More timely advice around the signs of readiness alongside the risks associated with early initiation will enable parents to make a more informed judgement around when to introduce solid foods. Moreover, advice should also acknowledge the influential roles of other family caregivers to encourage healthy feeding practices.

Through listening directly to the parents who have recently fed their infants, we have been able to learn about the common and distinct similarities and differences of cultural feeding attitudes and practices among culturally diverse and socially disadvantaged parents. These insights can help facilitate the questioning and tailoring of advice regarding infant feeding practices among health care professionals when working with parents. The rich and valuable insights can also facilitate the development of public health interventions that can build on the barriers revealed and empower parents to develop healthy and enjoyable eating habits in their infants.

## CONFLICTS OF INTEREST

The authors declare that they have no conflicts of interest.

## CONTRIBUTIONS

All authors were involved in conception and design of the study. All authors were involved in the fieldwork. EC, FP and CP‐J completed the qualitative data analysis. All authors contributed to interpretation and prioritisation of findings. EC drafted the paper. GR is the guarantor. All authors contributed to the manuscript and approved the submitted copy for publication.

## Footnotes

^1^
British (or English) cuisine is traditionally known for being meat and potato based, which includes dishes such as fish and chips, roast dinners, pies, pastries and bangers (sausages) and mash. Britain diet has developed to become more reflective of the cultural influences of its post‐colonial territories, in particular, South Asia. In more recent times, Britain has adopted a more westernised diet, shown to have the highest consumption of processed food in Europe (Monteiro et al., 2018).
